# Advancing Personalized Nutrition Through Genetic Nutritional Insights

**DOI:** 10.3390/nu17132166

**Published:** 2025-06-29

**Authors:** Michaela Marie St Germain, Amirreza Mortazavi, Marica Bakovic

**Affiliations:** Marica Bakovic’s Laboratory, Human Health and Nutritional Sciences Department, University of Guelph, 50 Stone Rd. E, Guelph, ON N1G 2W1, Canada; mstg0001@student.monash.edu (M.M.S.G.); amortaza@uoguelph.ca (A.M.)

Metabolic health is shaped by the dynamic interplay between genetic predispositions and dietary habits. As our understanding of this relationship deepens, we move closer to a nutritional framework that is not only evidence-based, but also personalized to each individual’s genetic profile. This Special Issue of *Nutrients* presents nine studies that explore gene–diet interactions across a range of health outcomes, highlighting both the promise and the complexity of nutrigenomics.

Given its strong links to both diet and genetic susceptibility, metabolic disease is a particularly relevant focus within nutrigenomics. This was addressed in the work of Oh et al. (2023) [contribution 1], which found that variations in the PNPLA3 gene significantly modulate non-alcoholic fatty liver disease (NAFLD) susceptibility, and that this risk was reduced in individuals with increased kimchi intake. This points to a potential role for fermented foods in the prevention of NAFLD in individuals with genetic predispositions. Such findings exemplify how personalized dietary guidance, shaped by genetic screening, could offer substantial health benefits by mitigating genetic risks through specific nutritional strategies. 

Expanding beyond NAFLD, Park (2023) [contribution 2] examined height-related polygenic variants, linking them to metabolic syndrome risk. Interestingly, taller individuals with specific genetic profiles had a reduced risk of metabolic syndrome, especially when consuming diets lower in rice and higher in energy. This underscores the influence of genetics on dietary needs and raises important considerations for culturally appropriate dietary recommendations. The study also investigated functional gene–diet interactions, showing that the GDF5_rs224331 variant alters how its protein product binds hydrolysable tannins—compounds known to support bone health. These findings illustrate how genetic variation shapes nutrient responses and can inform personalized strategies for managing chronic conditions like osteoporosis.

In addition to osteoporosis, this Special Issue includes novel insights into the genetic regulation of liver, cardiovascular, and thyroid disease. Ghare et al. (2023) [contribution 3] demonstrated that tributyrin, a butyrate precursor found in dairy and legumes, reduced alcohol-induced liver inflammation by reversing harmful epigenetic changes. This work not only highlights a therapeutic avenue for alcohol-associated liver disease, but also exemplifies how nutrient-derived compounds can directly modulate gene expression. Additionally, the study by Garrido-Sanchez et al. (2024) [contribution 4] sheds light on the connection between single nucleotide variants in the ABCG8 gene and phytosterol metabolism, which has associations with parenteral nutrition-associated liver disease and cardiovascular disease risk. Furthermore, Kim and Park (2023) [contribution 5] revealed significant interactions between polygenic variants related to hypothyroidism and lifestyle factors, including dietary habits and smoking. Such genetic–diet interactions offer promising pathways for personalized nutrition interventions aimed at reducing the incidence and severity of hypothyroidism, further demonstrating the vast potential for diet to modulate genetic risk.

Beyond disease risk, genetics can also influence dietary behaviors. Franzago et al. [contribution 6] (2023) found that variants in the CD36 gene—implicated in fat taste perception—were associated with differences in anthropometric and metabolic outcomes among individuals with diabetes or dysglycemia. These findings raise the possibility that sensory genetics could help tailor dietary counseling to improve adherence and outcomes.

Early-life nutrition and epigenetic mechanisms add another critical dimension to gene–diet interactions, as nutritional exposures during sensitive developmental periods can influence long-term gene expression through processes like DNA methylation. Patel et al. (2023) [contribution 7] highlighted the significance of dietary methyl donors such as folate, demonstrating their impact on methylation patterns of obesity-related genes like NRF1, FTO, and LEPR in children. This study illustrates the promise of early nutritional intervention but also the need for future research that accounts for intersecting biological and social determinants of health.

The study by Buchanan et al. (2024) [contribution 8] also investigated how nutrition during developmental periods can impact future disease using a mouse model of lifelong dietary exposure to n-3 polyunsaturated fatty acids (PUFAs). Their findings suggest that early n-3 PUFA intake, particularly during puberty, promotes mammary gland differentiation and reduces pro-carcinogenic gene expression patterns, pointing to the preventive potential of diet during sensitive developmental windows.

While many of the studies in this Special Issue report promising associations between genetics and dietary responses, it is equally important to acknowledge null findings that refine our understanding of where gene–diet interactions may or may not be significant. For instance, Górczyńska-Kosiorz et al. (2024) [contribution 9] finds no significant relationship between the obesity-related FTO gene, dietary patterns, and metabolic syndrome in a subset of young, healthy Polish men. However, the absence of significant findings itself reinforces a key point that genetic risk does not guarantee disease. Factors such as culture, environment, and protective behaviors can buffer genetic predisposition.

Overall, this Special Issue of *Nutrients* covers a wide range of topics in nutrigenetics and nutrigenomics; however, the overall message is clear. Diet and genetics are interdependent, and their impacts on chronic disease cannot be considered separately. These integrated findings from recent studies strongly advocate a shift towards precision nutrition, which leverages comprehensive genetic and epigenetic insights. In order to facilitate this transition, it is crucial to take the findings from observational and animal-based studies and transitions to clinical research. For example, based on the success of tributyrin supplementation in mouse models of alcohol-related liver disease, it could be reasonable to move toward a randomized controlled trial in humans. Furthermore, it would be interesting to explore the mechanisms behind gene and diet associations to gain a deeper understanding of the physiological impacts of nutrients. Namely, it would be interesting to explore potential causation and correlation between kimchi consumption and reduced risk of NAFLD in people with genetic predispositions, as this might be extrapolated to other fermented foods, as well as other chronic disease.

Finally, with all of this in mind, the feasibility of personalized, gene-based dietary counseling must be considered. This will require further research into the ethical, economic, and logistical challenges of implementation, including patient privacy, socioeconomic disparities, healthcare infrastructure, and the translation of genetic data into clinical practice. As nutrigenomics continues to evolve, future research must not only validate gene–diet interactions through clinical trials, but also investigate how best to integrate these insights into equitable, accessible, and effective public health strategies. The question now is not whether personalized nutrition is possible, but how we can responsibly and rigorously build the evidence to support its widespread, real-world application.

